# The Application of artificial intelligence in restorative Dentistry: A narrative review of current research

**DOI:** 10.1016/j.sdentj.2024.03.017

**Published:** 2024-03-21

**Authors:** Bilal Arjumand

**Affiliations:** Department of Conservative Dentistry, College of Dentistry, Qassim University, Saudi Arabia

**Keywords:** Artificial intelligence, Diagnosis, Restorative materials, Bio materials, Aesthetics

## Abstract

This review explores the transformative impact of artificial intelligence (AI) on restorative dentistry. By discussing the diagnostic processes, treatment planning, image analysis, prosthodontics, and material/biomaterial research, this study highlights the role of AI in optimizing precision and efficiency. It emphasizes personalized material selection, accelerated biomaterial research, and AI-enabled clinical workflows for enhanced patient outcomes. The review concludes with insights into the challenges, ethical considerations, and future trends, emphasizing the collaborative efforts needed for continued innovation in AI-driven restorative dentistry.

## 1 Introduction

**Fig. 1:**
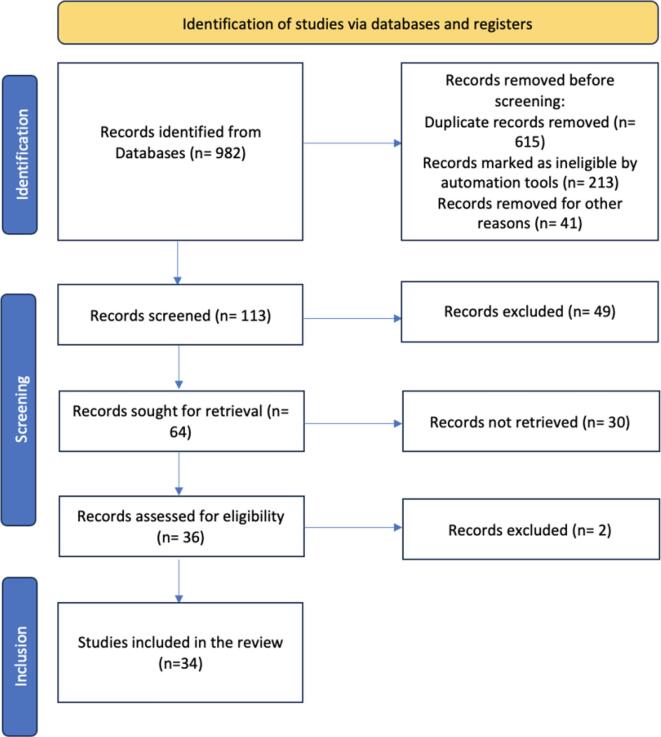
Flowchart showing the step-by-step identification of the studies from the databases.

Restorative dentistry, which is an integral component of oral healthcare, is primarily concerned with the restoration and maintenance of tooth structures (Kilpatrick et al., 2020). This sector is being redefined through a combination of cutting-edge technology and conventional practices. The increasing prominence of artificial intelligence (AI) in the healthcare industry is one indicator of this transition. To improve accuracy, efficiency, and patient outcomes, dentistry—which hitherto relied on human dexterity and experience—is increasingly using AI (Hussein N et al., 2022).Fig. 1Table 1.

**Table 1:** Assistance of AI in clinical workflow.

Author Names	**AI Application**	**Enhanced Decision-Making**	**Workflow Optimization**	Patient Outcomes Improvement
Urban R et al., 2023	Streamlining administrative tasks in clinical settings.	Automation of administrative tasks for efficiency.	Informed decision-making based on data analysis and insights.	Reduction of administrative burdens through AI-driven processes.
Tuzova L et al., 2023	Integrating AI for data analysis and decision support.	Data analysis for informed decision-making and insights.	Decision support for clinical interventions.	Integration of AI for real-time data analysis, leading to better patient outcomes.
Metsälä E et al., 2014	Enhancing diagnostic accuracy through AI-assisted interpretation of diagnostic tests and medical imaging.	Improving diagnostic accuracy through AI-assisted interpretation.	Automation of repetitive tasks to reduce errors.	Increased efficiency in healthcare delivery.
Suwardi A et al., 2022	Real-time monitoring for proactive healthcare.	Real-time monitoring for proactive patient care.	Improved collaboration and communication among healthcare professionals.	Patient-centric approach in clinical decision-making.
Benetti AR et al., 2019	Enriching clinical decision-making with AI-powered insights.	AI-powered insights for enhanced clinical decision-making.	Improved patient care coordination.	Improved patient satisfaction and adherence.
Yilmaz EC et al., 2020	Personalizing patient care with AI-driven recommendations.	AI-driven recommendations for personalized patient care.	Streamlined patient flow and reduced wait times.	Improved patient engagement and outcomes.

Abbreviation: AI, artificial intelligence.

Restorative dentistry is vital not only for its aesthetic benefits but also for its essential function in preserving tooth structures. AI is transforming the diagnosis and treatment of dental disorders owing to its ability to handle large datasets and identify complex patterns (Patil S et al., 2022). With the changing healthcare environment, the integration of AI into restorative dental operations is no longer only an alternative but rather a requirement to advance the industry.

The primary objective of this study was to comprehensively analyze the transformational influence of AI on restorative dentistry. This study examined the complexities of AI applications and investigated their potential in enhancing diagnostic processes, treatment planning, image analysis, and prosthodontics. Through an analysis of the present state of affairs, this study aimed to highlight the need for a reciprocal relationship between AI and dental practices.

## 2 Methodology

To conduct a literature survey on AI in restorative dentistry, a search was conducted in August 2023 across various electronic databases including PubMed, SCOPUS, EMBASE, the COCHRANE Library, and ScienceDirect. The search utilized MeSH terms/keywords such as “Dentistry,” “Artificial Intelligence,” “Restorative,” etc. In addition to the electronic searches, cross-references and textbooks were manually searched for relevant articles. The inclusion criteria were articles published in English between August 2000 and August 2023 that fulfilled the objectives of the study. The article selection process involved assessing the inclusion and exclusion criteria and conducting a quality assessment. Of the initially identified 982 articles, 113 were selected based on their titles and abstracts. Additionally, four articles were obtained through a manual search, resulting in a total of 117 articles. After evaluating the full texts and applying the inclusion and exclusion criteria, 34 articles were chosen for the review, meeting the study's criteria.

## 3 AI fundamentals in dentistry

### 3.1 Basics of AI

AI serves as the technological backbone reshaping the landscape of dentistry.

**Machine Learning (ML):** At the core of AI, machine learning involves algorithms that enable systems to learn and make predictions or decisions without explicit programming. In restorative dentistry, ML algorithms analyze vast datasets comprising patient records, diagnostic images, and treatment outcomes. This analysis facilitates predictive modelling for conditions such as periodontal diseases, aiding in early intervention and personalized treatment plans (Revilla-León M et al., 2022).

**Deep Learning (DL):** A subset of machine learning, deep learning employs neural networks inspired by the structure of the human brain. In dentistry, DL excels in image analysis and enhances diagnostics through the interpretation of radiographs and intraoral images. Its ability to identify patterns and anomalies contributes to more accurate and efficient diagnosis, which is a crucial aspect of restorative dental procedures (Rodrigues JA et al., 2021).

**Natural Language Processing (NLP):** Dentistry involves extensive record-keeping and communication. NLP enables machines to comprehend and generate human-like text and streamline tasks, such as patient history documentation and automated communication. This not only saves time for dental professionals but also ensures comprehensive patient records, contributing to more informed restorative dentistry decisions (Pethani F et al., 2023).

The relevance of these AI facets in restorative dentistry lies in their collective ability to swiftly and accurately analyze complex data. For example, ML algorithms can aid in predicting the success of dental implants based on patient-specific factors, whereas DL algorithms can enhance the interpretation of 3D images for precise prosthodontic procedures. NLP contributes to efficient communication, reducing the risk of misunderstandings in treatment planning.

### 3.2 AI tools and techniques

**Fig. 2:**
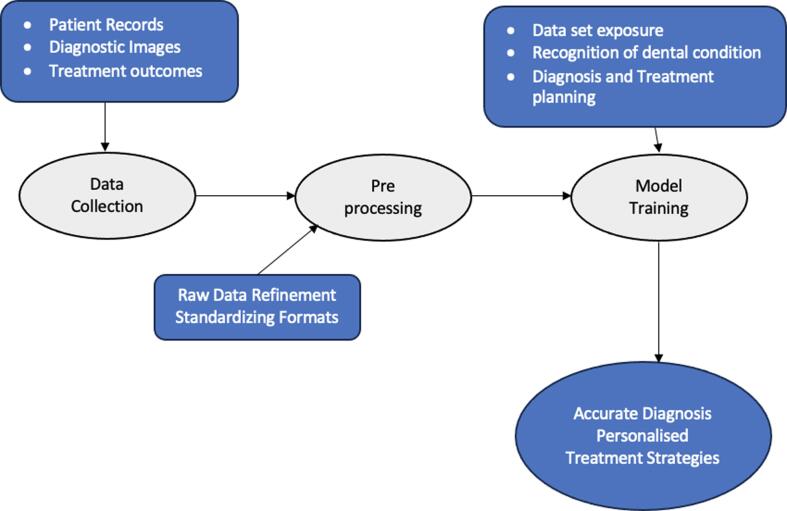
AI Tools and Techniques in Restorative Dentistry.

In the realm of dental applications, several AI tools and techniques are instrumental in advancing restorative dentistry (Fig. 2).

**Data Collection:** AI thrives on data; in dentistry, this encompasses patient records, diagnostic images, and historical treatment outcomes. The integration of electronic health records and imaging databases allows AI systems to access a wealth of information that is crucial for developing predictive models and treatment plans (Schwendicke FA et al., 2020).

**Pre-processing:** Raw data often require refinement for effective AI utilization. Preprocessing techniques involve cleaning and organizing data to enhance its quality. In restorative dentistry, this may involve standardized imaging formats, ensuring uniformity for accurate analysis (Ahmed N et al., 2021).

**Model Training:** Training AI models involves exposing them to vast datasets, thereby allowing them to learn patterns and correlations. In restorative dentistry, this training process is fundamental for developing algorithms capable of recognizing specific dental conditions and aiding in diagnosis and treatment planning (Shan T et al., 2021).

By comprehending these tools and techniques, dental professionals can harness the potential of AI to optimize restorative dentistry processes from accurate diagnostics to personalized treatment strategies.

## 4 Applications of AI in restorative dentistry

AI has become an indispensable tool in restorative dentistry, revolutionizing diagnostic processes, treatment planning, and fabrication of dental prostheses.

### 4.1 Diagnosis and treatment planning

The prowess of AI in analyzing extensive datasets and recognizing patterns makes it invaluable for diagnosing dental conditions and tailoring treatment plans. Applications of AI in diagnosis include the early identification of conditions like periodontal diseases and caries. Machine learning algorithms, when provided with patient data, clinical histories, and diagnostic images, can discern subtle patterns that are indicative of these conditions. Early detection allows for timely intervention, prevention of disease progression, and facilitation of less invasive treatment options (Asiri AF et al., 2022).

Treatment planning benefits significantly from the ability of AI to process vast amounts of patient data. AI can generate personalized treatment plans by considering individual health records, risk factors, and treatment outcomes. This personalized approach enhances treatment effectiveness and patient satisfaction, aligning with the shift towards precision medicine in dentistry (Agrawal P et al., 2022).

### 4.2 Image analysis

The impact of AI on image analysis in dentistry has been transformative, particularly concerning radiographs, CT scans, and intraoral images.

**Radiographs and CT Scans:** AI excels at interpreting radiographic images, aiding in the identification of dental pathologies with enhanced accuracy. Image segmentation techniques powered by deep learning algorithms enable the precise delineation of anatomical structures, allowing for better visualization of dental issues. Additionally, AI contributes to feature extraction, helping identify subtle changes indicative of diseases (De Angelis F et al., 2022).

**Intraoral Images:** The role of AI extends to intraoral images, where it aids in the detection of conditions such as enamel erosion, gingival inflammation, and early stage lesions. Image analysis algorithms can detect minute changes that may escape the human eye and contribute to comprehensive diagnostics (Kühnisch et al., 2022 Feb, Moharrami et al., 2023).

Advancements in image analysis not only improve diagnostic accuracy, but also expedite the process. Faster and more precise image interpretation reduces the time that patients spend in the diagnostic phase, facilitating quicker treatment initiation.

### 4.3 Prosthodontics and CAD/CAM

AI-driven advancements in Computer-Aided Design and Manufacturing (CAD/CAM) have reshaped the landscape of prosthodontics, offering benefits in terms of precision and efficiency (Yamaguchi S et al., 2019).

**Precision:** AI algorithms optimize the design of dental prostheses to ensure precise fit and functionality. Considering patient-specific anatomical variations and occlusal dynamics, AI contributes to the creation of prosthetic devices that closely mimic natural dentition. This level of precision minimizes complications and enhances the longevity of the restorations (Ding H et al., 2023).

**Efficiency:** AI-guided CAD/CAM processes are more efficient for streamlining the fabrication of dental prostheses. AI algorithms facilitate rapid prototyping, thereby reducing the time that patients spend in the prosthodontic treatment cycle. Moreover, the iterative nature of AI allows for continuous refinement, contributing to improvements in the overall efficiency of CAD/CAM systems (ALBAYRAK B et al.,2021).

## 5 AI in restorative materials and biomaterials

### 5.1 Material selection

AI has revolutionized restorative dentistry by being integrated into the critical material selection process. The use of AI in this particular situation surpasses traditional practices by providing a customized strategy that relies on patient-specific data (El Gezawi M et al., 2019).

**Personalization Through Data Analysis:** By analyzing patient data, AI considers a wide range of details, including medical history, lifestyle, and individual oral characteristics (Höland W et al., 2008). The thorough examination presented in Höland et al. allowed a more nuanced comprehension of the distinct needs of each patient, thus affecting the choice of materials in accordance with their particular demands.

**Optimization for Longevity and Aesthetics:** AI systems analyze extensive datasets, detecting associations between the efficacy of various materials and their durability within certain patient populations. AI plays a significant role in facilitating the attainment of ideal aesthetic results by examining past data pertaining to patient preferences and reactions to various materials (McCabe JF et al., 2004).

**Predictive Modelling:** The use of AI's predictive modelling skills is of significant importance in the process of material selection. By examining the performance of different materials in a range of therapeutic circumstances, AI can predict the prospective efficacy of a certain substance in a given patient. The use of predictive modelling techniques serves to diminish the dependence on trial-and-error methodologies, thus guaranteeing a more streamlined and proficient selection procedure. (Basu B et al., 2022).

**Real-time Decision Support:** AI plays a crucial role as a real-time decision assistance system for dental practitioners throughout the material selection process. AI enables dentists to make informed judgments consistent with evidence-based practices that are tailored to specific patient profiles. This is achieved via the utilization of up-to-date research and patient data, which AI promptly analyzes to provide immediate insights and suggestions (Benetti AR et al., 2019).

**Case-specific Considerations:** Every case in the field of restorative dentistry has distinct characteristics, and AI demonstrates exceptional proficiency in comprehending the complexities associated with each case. Regardless of the complexity of the restoration, an evaluation conducted using AI considers several factors including occlusion, load-bearing needs, and patient preferences. (Yilmaz EC et al., 2020).

### 5.2 Biomaterial research

The incorporation of AI has had a notable impact on the advancements in biomaterial research in restorative dentistry. This section explores the significant role of AI in driving innovation in the field of biomaterials, with particular emphasis on its capacity to enhance biocompatibility and durability.

**Accelerated Discovery Through AI:** Traditional biomaterial research processes are often time-consuming and involve extensive laboratory experimentation. AI expedites this journey by analyzing vast datasets encompassing material properties, patient responses, and clinical outcomes. By identifying patterns and correlations, AI accelerates the discovery of novel biomaterials with enhanced biocompatibility (Suwardi A et al., 2022).

**Biocompatibility Enhancement:** AI improves biocompatibility by discerning the intricate relationships between material characteristics and biological responses. This goes beyond the traditional understanding of biocompatibility and allows for the development of materials tailored to specific patient profiles. AI-driven biomaterials are designed not only to perform functionally but also to integrate seamlessly with the patient’s biological milieu (Tayebi L et al., 2017).

**Durability Optimization:** Durability is a critical factor in the success of restorative procedures. AI analyzes the wear and degradation patterns of different materials over time and predicts their long-term performance. This predictive capability enables the optimization of biomaterials for durability, ensuring that restorations can withstand the challenges of the oral environment and maintain their integrity over an extended period (Georgeanu VA et al., 2023).

**Adaptive Biomaterials:** The continuous learning capabilities of AI have contributed to the development of adaptive biomaterials. These materials dynamically respond to changes in the oral environment and adapt to their properties to ensure sustained performance. This adaptability is particularly valuable in scenarios in which oral conditions fluctuate, such as changes in pH or exposure to different types of stress (Ratner BD et al., 1996).

**Patient-Centric Biomaterial Design:** AI-driven biomaterial research is inherently patient-centric. By considering individual patient characteristics, such as genetic factors and immune responses, AI facilitates the design of biomaterials that are not only effective across diverse populations but also tailored to the specific needs of each patient (Parhi S et al., 2021).

## 6 Ai-enabled clinical workflow

### 6.1 Patient management and records

The incorporation of AI into the clinical workflow of dental practices revolutionizes patient management and record-keeping, ushering in unprecedented efficiency and precision (Urban R et al., 2023).

**Streamlining Administrative Tasks:** AI applications play a pivotal role in automating the routine administrative tasks associated with patient management. From appointment scheduling to updating treatment histories, AI systems streamline these processes, reducing the administrative burden on the dental staff. Intelligent scheduling algorithms optimize appointment slots, minimize patient wait times, and maximize dental resource utilization.

**Personalized Patient Records:** AI data analysis capabilities contribute to the creation of comprehensive and personalized patient records. By integrating information from various sources including electronic health records and patient-reported data, AI ensures that practitioners have a holistic view of each patient’s oral health. This personalized approach enhances treatment planning and allows for more informed decision-making.

**Predictive Analytics for Appointments:** AI uses predictive analytics to forecast patient appointment patterns. AI algorithms can predict periods of high demand or identify potential appointment cancellations by analyzing historical data, patient preferences, and external factors. This foresight aids in optimizing appointment schedules and ensuring the efficient use of clinic resources.

**Enhanced Communication Channels:** AI-powered communication systems facilitate seamless interactions between dental practitioners and patients. For example, chatbots can provide instant responses to common queries, schedule appointments, and send automated reminders. This not only improves patient engagement, but also frees up staff time for more complex interactions.

**Security and Compliance:** AI ensures the security and compliance of patient records. Advanced encryption algorithms safeguard sensitive information, and AI-driven systems assist in maintaining compliance with healthcare regulations, such as the Health Insurance Portability and Accountability Act (HIPAA).

### 6.2 Quality assurance and monitoring

Quality assurance and real-time monitoring during restorative procedures are critical for ensuring successful outcomes. AI has introduced a paradigm shift in this domain, offering advanced tools for continuous assessment and error prevention.

**Real-time Feedback Mechanisms:** AI-enabled systems provide real-time feedback to practitioners during restoration. By analyzing live data from various diagnostic tools and imaging devices, AI can highlight potential issues, deviations from treatment plans, and areas requiring additional attention. This immediate feedback enhances the precision of the procedures and reduces the likelihood of errors (Tuzova L et al., 2023).

**Error Prevention and Intervention:** AI acts as a proactive guardian against procedural errors. Machine learning algorithms trained on extensive datasets of successful and unsuccessful cases can identify patterns indicative of potential errors. In the event of detected anomalies, the system can trigger alerts or suggest corrective actions, thereby enabling prompt intervention and mitigating the risk of complications.

**Post-Procedure Monitoring:** After a restorative procedure, AI continues to play a role in monitoring patient outcomes. AI contributes to the ongoing assessment of restoration success by analyzing post-procedural data, including patient-reported symptoms and follow-up diagnostic imaging. Continuous monitoring allows for timely intervention if issues arise.

**Quality Control in Prosthodontics:** In prosthodontics, where precision is paramount, AI-driven quality control mechanisms ensure that manufactured dental prostheses meet specified standards. Computer-aided design and manufacturing (CAD/CAM) processes guided by AI algorithms enhance the accuracy of prosthetic restorations, minimizing discrepancies and optimizing the fit (Metsälä E et al., 2014).

## 7 Enhancing aesthetic outcomes in restorative dentistry with AI

Incorporating AI into aesthetic design and treatment planning has revolutionized restorative dentistry. AI tools are pivotal for analyzing facial symmetry and patient-specific features, aiding comprehensive aesthetic evaluations. They play a crucial role in developing personalized treatment plans that prioritize aesthetic outcomes, including tooth color and alignment. Moreover, the ability of AI to simulate and predict preprocedural aesthetic results through advanced 3D modelling is transformative, enabling patients and dentists to visualize potential outcomes. Additionally, the application of AI extends to material selection and the design of dental prosthetics, ensuring that each restoration not only fits functionally, but also complements the patient’s overall appearance. This integration of AI promises to increase the standards of esthetic satisfaction in restorative dental treatments.

## 8 Challenges and ethical considerations

### 8.1 Data privacy and security

In the era of AI-driven dentistry, safeguarding patient data privacy and ensuring security are paramount. The seamless integration of AI into dental practice relies heavily on the collection, processing, and analysis of sensitive patient information. To emphasize the importance of data privacy, dental practitioners must adhere to stringent healthcare regulations such as the Health Insurance Portability and Accountability Act (HIPAA) (Joda T et al., 2019). Compliance ensures that patient data are handled with utmost confidentiality and security, preventing unauthorized access or data breaches. AI developers and dental professionals must collaborate to implement robust encryption protocols, access controls, and audit trails to fortify defense against potential cyber threats.

### 8.2 Training and education

The integration of AI technology into dentistry necessitates a paradigm shift in the training and education of dental professionals. Although AI promises transformative benefits, challenges arise in ensuring that practitioners are proficient at effectively leveraging these technologies. Dental curricula must incorporate comprehensive training programs that cover the fundamentals of AI, its applications in dentistry, and hands-on experience with AI-driven tools (Huang YK et al., 2022). Overcoming potential resistance to adopting new technologies requires concerted efforts from educational institutions, dental associations, and practitioners. Continuous professional development should be encouraged to keep dental professionals abreast of evolving AI applications and best practices.

### 8.3 Ethical and legal issues

As AI has become an integral part of restorative dentistry, its ethical considerations and legal implications merit careful attention. Ethical dilemmas may arise concerning issues such as informed consent, transparency in AI decision-making processes, and the responsible use of patient data. Dental practitioners bear the ethical responsibility of communicating effectively with patients about AI-assisted procedures and ensuring that they understand the role of the technology in their treatment (Mörch CM et al., 2021). Legal frameworks should evolve to address the unique challenges posed by AI applications in dentistry and define the responsibilities of both dental practitioners and AI developers. Striking the right balance between innovation and ethical practice is essential to foster trust and ensure the ethical use of AI in the field.

## 9 Future directions and emerging trends

The future of AI in restorative dentistry holds exciting possibilities and trends. Enhanced diagnostic accuracy, personalized treatment plans, and the development of advanced biomaterials are anticipated (Surlari et al., 2023). AI-driven robot-assisted surgeries, further integration of AI into chairside procedures, and use of AI in preventive dentistry are emerging trends. The collaborative efforts of dental professionals and AI experts are likely to lead to innovative applications and transform the landscape of restorative dentistry.

## 10 Conclusion

In conclusion, the integration of AI into restorative dentistry represents a significant leap forward in enhancing patient care, diagnostic accuracy, and treatment outcomes. As AI technologies continue to advance, dental practitioners must navigate the challenges related to data privacy, embrace comprehensive training, and uphold ethical standards. Collaborative synergy between dental professionals and AI experts is crucial for unlocking the full potential of AI in advancing restorative dentistry. Encouraging further research, dialogue, and collaboration will foster a dynamic and innovative future in which AI will contribute seamlessly to the evolution of dental healthcare.

Ethical Statement

Hereby, I Dr. Bilal Arjumand consciously assure that for the manuscript: The Application of Artificial Intelligence in Restorative Dentistry: A Narrative Review of Current Research“ the following is fulfilled:

1) This material is the authors' own original work, which has not been previously published elsewhere.
2) The paper is not currently being considered for publication elsewhere.
3) The paper reflects the authors' own research and analysis in a truthful and complete manner.
4) All sources used are properly disclosed (correct citation). Literally copying of text must be indicated as such by using quotation marks and giving proper reference.
5) Author personally and actively involved in substantial work leading to the paper, and will take public responsibility for its content.

I Agree with the above statements and declare that this submission follows the ethical policies

## Declaration of Competing Interest

The authors declare that they have no known competing financial interests or personal relationships that could have appeared to influence the work reported in this paper.
