# Supplementary material for: The Application of artificial intelligence in restorative Dentistry: A narrative review of current research
Source: Saudi Dent J. 2024 Mar 21;36(6):835–40. doi: 10.1016/j.sdentj.2024.03.017 (PMC11178959; doi:10.1016/j.sdentj.2024.03.017)
Supplement: Supplementary data 1 [file mmc1.pdf]

ELSEVIER

Certificate of Elsevier Language Editing Services

The following article was edited by Elsevier Language Editing Services:

The Application of Artificial Intelligence in Restorative Dentistry: A Narrative Review of Current Research

Ordered by:

Bilal Arjumand

Estimated Delivery date:

2024-02-12

Order reference:

ASLESTD1043593
