# Supplementary material for: The Application of artificial intelligence in restorative Dentistry: A narrative review of current research
Source: Saudi Dent J. 2024 Mar 21;36(6):835–40. doi: 10.1016/j.sdentj.2024.03.017 (PMC11178959; doi:10.1016/j.sdentj.2024.03.017)
Supplement: Supplementary data 2 [file mmc2.pdf]

AI in restorative dentisrty

by Dr Bilal Review

Submission date: 22-Nov-2023 03:56PM (UTC+0400)
Submission ID: 2236078261
File name: tion_of_Artificial_Intelligence_in_Restorative_Dentistry_1.docx (323.76K)
Word count: 4282
Character count: 27670

The Application of Artificial Intelligence in Restorative Dentistry: A Narrative Review of Current Research

Abstract

This review explores the transformative impact of artificial intelligence (AI) on restorative dentistry. Covering diagnostic processes, treatment planning, image analysis, prosthodontics, and material/biomaterial research, the study highlights AI's role in optimising precision and efficiency. It emphasises personalised material selection, accelerated biomaterial research, and AI-enabled clinical workflows for enhanced patient outcomes. The review concludes with insights into challenges, ethical considerations, and future trends, emphasising the collaborative efforts needed for continued innovation in AI-driven restorative dentistry.

1. Introduction

Restorative dentistry, an integral component of oral healthcare, is primarily concerned with the restoration and maintenance of tooth structures (Kilpatrick et al., 2020). This sector is being redefined by the combination of cutting-edge technology and conventional practices. The increasing prominence of artificial intelligence (AI) in the healthcare industry is one indicator of this transition. In order to improve accuracy, efficiency, and patient outcomes, dentistry—which hitherto relied on human dexterity and experience—is increasingly using artificial intelligence (Hussein N et al., 2022).

Restorative dentistry is vital not just for its aesthetic benefits, but also for its essential function in preserving tooth structures. Artificial intelligence (AI) is transforming the diagnosis and treatment of dental disorders with its ability to handle enormous datasets and spot complex patterns (Patil S et al., 2022). With the changing healthcare environment, the integration of artificial intelligence (AI) into restorative dental operations is no longer only an alternative but rather a need to advance the industry.

The primary objective of this study is to provide a comprehensive analysis of the transformational influence of artificial intelligence (AI) on the field of restorative dentistry. This research examines the complexities of artificial intelligence (AI) applications, investigating its potential in enhancing diagnostic processes, treatment planning, image analysis, and prosthodontics. Through an analysis of the present state of affairs, this research aims to highlight the need of a reciprocal relationship between artificial intelligence (AI) and dentistry practices.

2. Methodology

To conduct a literature survey on Artificial Intelligence in Restorative Dentistry, a search was conducted in August 2023 across various electronic databases, including PubMed, SCOPUS, EMBASE, COCHRANE Library, and ScienceDirect. The search utilised MeSH terms/keywords such as "Dentistry," "Artificial Intelligence," "Restorative," etc. In addition to the electronic searches, cross-references and textbooks were manually searched for relevant articles. The inclusion criteria included articles published in English between August 2000 and August 2023 that fulfilled the study's objectives. The article selection process involved assessing the inclusion and exclusion criteria, as well as conducting a quality assessment. Out of the initially identified 982 articles, 113 were selected based on their titles and abstracts. Additionally, four articles were obtained through manual searching, resulting in a total of 117 articles. After evaluating the full texts and applying the inclusion and exclusion criteria, 34 articles were chosen for the review, meeting the study's criteria.

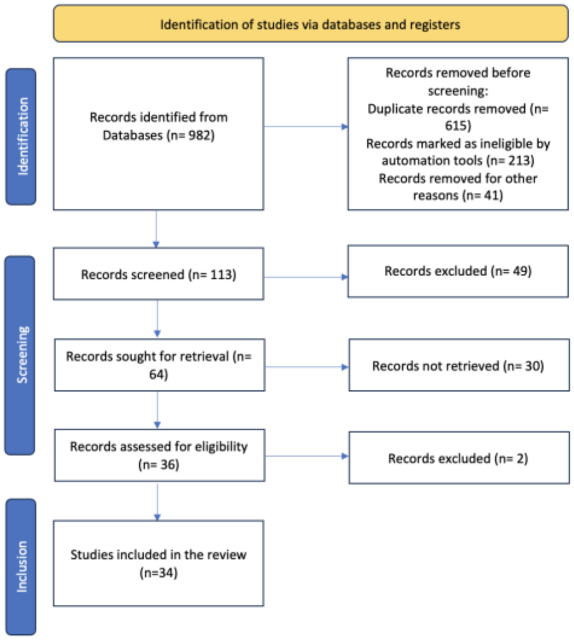

Figure 1: Flowchart showing the step-by-step identification of the studies via databases

3. AI Fundamentals in Dentistry

3.1 Basics of Artificial Intelligence

Artificial Intelligence (AI) serves as the technological backbone reshaping the landscape of dentistry.

Machine Learning (ML): At the core of AI, machine learning involves algorithms that enable systems to learn and make predictions or decisions without being explicitly programmed. In restorative dentistry, ML algorithms analyse vast datasets comprising patient records, diagnostic images, and treatment outcomes. This analysis facilitates predictive modelling for conditions such as periodontal diseases, aiding in early intervention and personalised treatment plans (Revilla-León M et al., 2022).

Deep Learning (DL): A subset of machine learning, deep learning employs neural networks inspired by the human brain's structure. In dentistry, DL excels in image analysis, enhancing diagnostics through the interpretation of radiographs and intraoral images. Its ability to identify

patterns and anomalies contributes to more accurate and efficient diagnosis, a crucial aspect in restorative dental procedures (Rodrigues JA et al., 2021).

Natural Language Processing (NLP): Dentistry involves extensive record-keeping and communication. NLP enables machines to comprehend and generate human-like text, streamlining tasks such as patient history documentation and automated communication. This not only saves time for dental professionals but also ensures comprehensive patient records, contributing to more informed restorative dentistry decisions (Pethani F et al., 2023).

The relevance of these AI facets to restorative dentistry lies in their collective ability to analyse complex data swiftly and accurately. For instance, ML algorithms can aid in predicting the success of dental implants based on patient-specific factors, while DL can enhance the interpretation of 3D imaging for precise prosthodontic procedures. NLP contributes to efficient communication, reducing the risk of misunderstandings in treatment planning.

3.2 AI Tools and Techniques

In the realm of dental applications, several AI tools and techniques are instrumental in advancing restorative dentistry (Figure 2).

Data Collection: AI thrives on data, and in dentistry, this encompasses patient records, diagnostic images, and historical treatment outcomes. The integration of electronic health records and imaging databases allows AI systems to access a wealth of information crucial for developing predictive models and treatment plans (Schwendicke FA et al., 2020).

Pre-processing: Raw data often requires refinement for effective AI utilisation. Pre-processing techniques involve cleaning and organising data to enhance its quality. In restorative dentistry, this may involve standardising imaging formats, ensuring uniformity for accurate analysis (Ahmed N et al., 2021).

Model Training: Training AI models involves exposing them to vast datasets, allowing them to learn patterns and correlations. In restorative dentistry, this training process is fundamental

for developing algorithms capable of recognising specific dental conditions, aiding in diagnosis, and treatment planning (Shan T et al., 2021).

By comprehending these tools and techniques, dental professionals can harness AI's potential to optimise restorative dentistry processes, from accurate diagnostics to personalised treatment strategies.

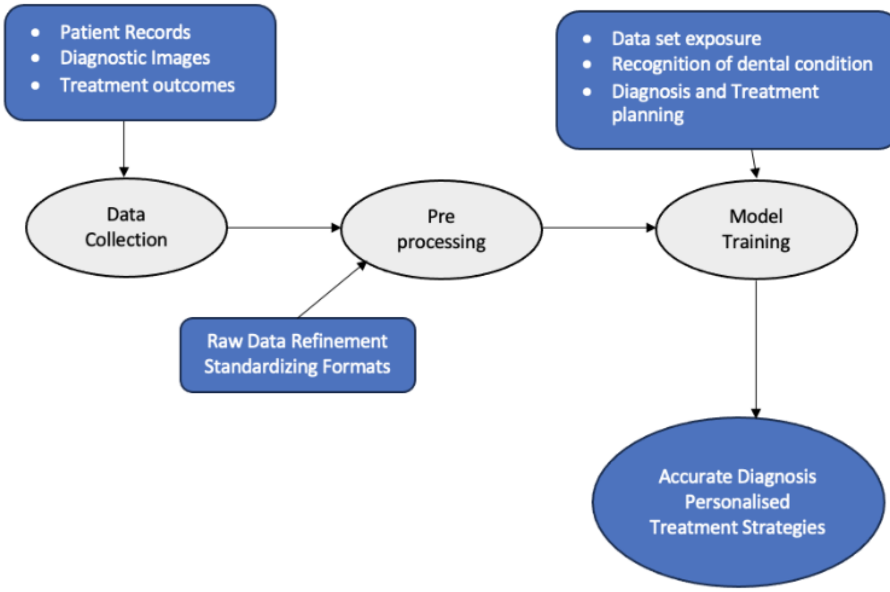

Figure 2: AI Tools and Techniques in Restorative Dentistry

20

4. Applications of AI in Restorative Dentistry

Artificial Intelligence (AI) has become an indispensable tool in restorative dentistry, revolutionising diagnostic processes, treatment planning, and the fabrication of dental prostheses.

4.1 Diagnosis and Treatment Planning

AI's prowess in analysing extensive datasets and recognising patterns makes it invaluable in diagnosing dental conditions and tailoring treatment plans. AI applications in diagnosis include the early identification of conditions like periodontal diseases and caries. Machine learning algorithms, when fed with patient data, clinical histories, and diagnostic images, can discern subtle patterns indicative of these conditions. Such early detection allows for timely interventions, preventing the progression of diseases and facilitating less invasive treatment options (Asiri AF et al., 2022).

Treatment planning benefits significantly from AI's ability to process vast amounts of patient data. By considering individual health records, risk factors, and treatment outcomes, AI can generate personalised treatment plans. This personalised approach enhances treatment effectiveness and patient satisfaction, aligning with the shift towards precision medicine in dentistry (Agrawal P et al., 2022).

4.2 Image Analysis

AI's impact on image analysis within dentistry is transformative, especially concerning radiographs, CT scans, and intraoral images.

Radiographs and CT Scans: AI excels in interpreting radiographic images, aiding in the identification of dental pathologies with enhanced accuracy. Image segmentation techniques, powered by deep learning algorithms, enable the precise delineation of anatomical structures, allowing for better visualisation of dental issues. Additionally, AI contributes to feature extraction, helping in the identification of subtle changes indicative of diseases (De Angelis F et al., 2022).

Intraoral Images: AI's role extends to intraoral images, where it aids in the detection of conditions like enamel erosion, gingival inflammation, and early-stage lesions. Image analysis algorithms can detect minute changes that might escape the human eye, contributing to comprehensive diagnostics (Kühnisch J et al., 2022; Moharrami M et al., 2023).

Advancements in image analysis not only improve diagnostic accuracy but also expedite the process. Faster and more precise image interpretation reduces the time patients spend in diagnostic phases, facilitating quicker treatment initiation.

4.3 Prosthodontics and CAD/CAM

AI-driven advancements in Computer-Aided Design and Manufacturing (CAD/CAM) have reshaped the landscape of prosthodontics, offering benefits in precision and efficiency (Yamaguchi S et al., 2019).

Precision: AI algorithms optimise the design of dental prostheses, ensuring a precise fit and functionality. By considering patient-specific anatomical variations and occlusal dynamics, AI contributes to the creation of prosthetic devices that closely mimic natural dentition. This level of precision minimises complications and enhances the longevity of restorations (Ding H et al., 2023).

Efficiency: CAD/CAM processes guided by AI are more efficient, streamlining the fabrication of dental prostheses. AI algorithms facilitate rapid prototyping, reducing the time patients spend in the prosthodontic treatment cycle. Moreover, the iterative nature of AI allows continuous refinement, contributing to improvements in the overall efficiency of CAD/CAM systems (ALBAYRAK B et al.,2021).

5. AI in Restorative Materials and Biomaterials

5.1 Material Selection

Artificial intelligence (AI) has revolutionised restorative dentistry by being integrated into the critical material selection process. The use of artificial intelligence (AI) in this particular situation surpasses traditional practises by providing a customised strategy that relies on patient-specific data (El Gezawi M et al., 2019).

Personalisation Through Data Analysis: By analysing patient data, AI may take into account a wide range of details, including medical history, lifestyle, and individual oral characteristics

(Höland W et al., 2008). The thorough examination presented in this study allows a more nuanced comprehension of the distinct needs of each patients, hence impacting the choice of materials that are in accordance with their particular demands.

Optimisation for Longevity and Aesthetics: Artificial intelligence systems analyse extensive datasets, detecting associations between the efficacy of various materials and their durability within certain patient populations. Artificial intelligence (AI) plays a significant role in facilitating the attainment of ideal aesthetic results by examining past data pertaining to patient preferences and their reactions to various materials (McCabe JF et al., 2004).

Predictive Modelling: The use of AI's predictive modelling skills is of significant importance in the process of material selection. Through the examination of the performance of different materials in a range of therapeutic circumstances, artificial intelligence (AI) has the capability to predict the prospective efficacy of a certain substance in a given patient. The use of predictive modelling techniques serves to diminish the dependence on trial-and-error methodologies, so guaranteeing a more streamlined and proficient choosing procedure. (Basu B et al., 2022).

Real-time Decision Support: AI plays a crucial role as a real-time decision assistance system for dental practitioners throughout the process of material selection. Artificial intelligence (AI) enables dentists to make well-informed judgements that are consistent with evidence-based practises and tailored to specific patient profiles. This is achieved via the utilisation of up-to-date research and patient data, which AI promptly analyses to provide immediate insights and suggestions. (Benetti AR et al., 2019).

Case-specific Considerations: Every individual case in the field of restorative dentistry has its own distinct characteristics, and artificial intelligence (AI) demonstrates exceptional proficiency in comprehending the complexities associated with each case. Regardless of the complexity of the restoration, the evaluation conducted by artificial intelligence (AI) takes into

account several factors, including occlusion, load-bearing needs, and patient preferences. (Yilmaz EC et al., 2020).

5.2 Biomaterial Research

The incorporation of artificial intelligence (AI) has had a notable impact on the advancement of biomaterial research in the field of restorative dentistry. This section explores the significant role of artificial intelligence (AI) in driving innovation in the field of biomaterials, with a particular emphasis on its capacity to enhance biocompatibility and durability.

Accelerated Discovery Through AI: Traditional biomaterial research processes are often time-consuming, involving extensive laboratory experimentation. AI expedites this journey by analysing vast datasets encompassing material properties, patient responses, and clinical outcomes. By identifying patterns and correlations, AI accelerates the discovery of novel biomaterials with enhanced biocompatibility (Suwardi A et al., 2022).

Biocompatibility Enhancement: AI contributes to the improvement of biocompatibility by discerning the intricate relationships between material characteristics and biological responses. This goes beyond the traditional understanding of biocompatibility, allowing for the development of materials tailored to specific patient profiles. AI-driven biomaterials are designed not only to perform functionally but also to integrate seamlessly with the patient's biological milieu (Tayebi L et al., 2017).

Durability Optimisation: Durability is a critical factor in the success of restorative procedures. AI analyses the wear and degradation patterns of different materials over time, predicting their long-term performance. This predictive capability enables the optimisation of biomaterials for durability, ensuring that restorations withstand the challenges of the oral environment and maintain their integrity over an extended period (Georgeanu VA et al., 2023).

Adaptive Biomaterials: AI's continuous learning capabilities contribute to the development of adaptive biomaterials. These materials can respond dynamically to changes in the oral

environment, adapting their properties to ensure sustained performance. This adaptability is particularly valuable in scenarios where the oral conditions may undergo fluctuations, such as changes in pH or exposure to different types of stresses (Ratner BD et al., 1996).

Patient-Centric Biomaterial Design: AI-driven biomaterial research is inherently patient-centric. By considering individual patient characteristics, such as genetic factors and immune responses, AI facilitates the design of biomaterials that are not only effective across diverse populations but also 5 tailored to the specific needs of each patient (Parhi S et al., 2021).

6. AI-Enabled Clinical Workflow

6.1 Patient Management and Records

The incorporation 17 of artificial intelligence (AI) into the clinical workflow of dental practices revolutionises patient management and record-keeping, ushering in unprecedented efficiency and precision (Urban R et al., 2023).

Streamlining Administrative Tasks: AI applications play a pivotal role in automating routine administrative tasks associated with patient management. From appointment scheduling to updating treatment histories, AI systems streamline these processes, reducing the administrative burden on dental staff. Intelligent scheduling algorithms optimise appointment slots, minimising wait times for patients and maximising the utilisation of dental resources.

Personalised Patient Records: AI's data analysis capabilities contribute to the creation of comprehensive and personalised patient records. By integrating information 10 from various sources, including electronic health records and patient-reported data, AI ensures that practitioners have a holistic view of each patient's oral health. This personalised approach enhances treatment planning and allows for more informed decision-making.

Predictive Analytics for Appointments: AI utilises predictive analytics to forecast patient appointment patterns. By analysing historical data, patient preferences, and external factors, AI algorithms can predict periods of high demand or identify potential appointment cancellations.

This foresight aids in optimising appointment schedules, ensuring efficient use of clinic resources.

Enhanced Communication Channels: AI-powered communication systems facilitate seamless interactions between dental practitioners and patients. Chatbots, for instance, can provide instant responses to common queries, schedule appointments, and send automated reminders. This not only improves patient engagement but also frees up staff time for more complex interactions.

Security and Compliance: AI contributes to ensuring the security and compliance of patient records. Advanced encryption algorithms safeguard sensitive information, and AI-driven systems assist in maintaining compliance with healthcare regulations, such as the Health Insurance Portability and Accountability Act (HIPAA).

6.2 Quality Assurance and Monitoring

Quality assurance and real-time monitoring during restorative procedures are critical aspects of ensuring successful outcomes. AI introduces a paradigm shift in this domain, offering advanced tools for continuous assessment and error prevention.

Real-time Feedback Mechanisms: AI-enabled systems provide real-time feedback to practitioners during restorative procedures. By analysing live data from various diagnostic tools and imaging devices, AI can highlight potential issues, deviations from the treatment plan, or areas requiring additional attention. This immediate feedback enhances the precision of procedures and reduces the likelihood of errors (Tuzova L et al., 2023).

Error Prevention and Intervention: AI acts as a proactive guardian against procedural errors. Machine learning algorithms, trained on extensive datasets of successful and unsuccessful cases, can identify patterns indicative of potential errors. In the event of detected anomalies, the system can trigger alerts or suggest corrective actions, enabling prompt intervention and mitigating the risk of complications.

Post-Procedure Monitoring: After a restorative procedure, AI continues to play a role in monitoring patient outcomes. By analysing post-procedural data, including patient-reported symptoms and follow-up diagnostic imaging, AI contributes to the ongoing assessment of the restoration's success. This continuous monitoring allows for timely interventions if any issues arise.

Quality Control in Prosthodontics: In prosthodontics, where precision is paramount, AI-driven quality control mechanisms ensure that manufactured dental prostheses meet the specified standards. Computer-aided design and manufacturing (CAD/CAM) processes guided by AI algorithms enhance the accuracy of prosthetic restorations, minimising discrepancies and optimising the fit (Metsälä E et al., 2014).

Table 1: Assistance of AI in clinical workflow

Author Names	AI Application	Enhanced Decision-Making	Workflow Optimization	Patient Outcomes Improvement
Urban R et al., 2023	Streamlining administrative tasks in clinical settings.	Automation of administrative tasks for efficiency.	Informed Decision-Making based on data analysis and insights.	Reduction of administrative burdens through AI-driven processes.
Tuzova L et al., 2023	Integrating AI for data analysis and decision support.	Data analysis for informed decision-making and insights.	Decision support for clinical interventions.	Integration of AI for real-time data analysis leading to better patient outcomes.
Metsälä E et al., 2014	Enhancing diagnostic accuracy through AI-assisted interpretation of diagnostic tests and medical imaging.	Improving diagnostic accuracy through AI-assisted interpretation.	Automation of repetitive tasks to reduce errors.	Increased efficiency in healthcare delivery.
Suwardi A et al., 2022	Real-time monitoring for proactive healthcare.	Real-time monitoring for proactive patient care.	Improved collaboration and communication among healthcare professionals.	Patient-centric approach in clinical decision-making.
Benetti AR et al., 2019	Enriching clinical decision-making with AI-powered insights.	AI-powered insights for enhanced clinical decision-making.	Improved patient care coordination.	Improved patient satisfaction and adherence.
Yilmaz EC et al., 2020	Personalising patient care with AI-driven recommendations.	AI-driven recommendations for personalised patient care.	Streamlined patient flow and reduced wait times.	Improved patient engagement and outcomes.

7. Challenges and Ethical Considerations

7.1 Data Privacy and Security

In the era of AI-driven dentistry, safeguarding patient data privacy and ensuring security are paramount. The seamless integration of artificial intelligence into dental practices relies heavily on the collection, processing, and analysis of sensitive patient information. Emphasising the importance of data privacy, dental practitioners must adhere to stringent healthcare regulations, such as the Health Insurance Portability and Accountability Act (HIPAA) (Joda T et al., 2019). Compliance ensures that patient data is handled with the utmost confidentiality and security, preventing unauthorised access or data breaches. AI developers and dental professionals must collaborate to implement robust encryption protocols, access controls, and audit trails to fortify the Defenses against potential cyber threats.

7.2 Training and Education

The integration of AI technology in dentistry necessitates a paradigm shift in the training and education of dental professionals. While AI promises transformative benefits, challenges arise in ensuring that practitioners are proficient in leveraging these technologies effectively. Dental curricula need to incorporate comprehensive training programs that cover the fundamentals of AI, its applications in dentistry, and hands-on experience with AI-driven tools (Huang YK et al., 2022). Overcoming the potential resistance to adopting new technologies requires a concerted effort from educational institutions, dental associations, and practitioners. Continuous professional development should be encouraged to keep dental professionals abreast of evolving AI applications and best practices.

7.3 Ethical and Legal Issues

As AI becomes an integral part of restorative dentistry, ethical considerations and legal implications merit careful attention. Ethical dilemmas may arise concerning issues such as informed consent, transparency in AI decision-making processes, and the responsible use of

patient data. Dental practitioners bear the ethical responsibility to communicate effectively with patients about AI-assisted procedures, ensuring they understand the technology's role in their treatment (Mörch CM et al., 2021). Legal frameworks should evolve to address the unique challenges posed by AI applications in dentistry, defining the responsibilities of both dental practitioners and AI developers. Striking the right balance between innovation and ethical practice is essential for fostering trust and ensuring the ethical use of AI in the field.

8. Future Directions and Emerging Trends

The future of AI in restorative dentistry holds exciting possibilities and trends. Enhanced diagnostic accuracy, personalised treatment plans, and the development of advanced biomaterials are anticipated. AI-driven robotic-assisted surgeries, further integration of AI into chairside procedures, and the use of AI in preventive dentistry are emerging trends. The collaborative efforts of dental professionals and AI experts are likely to lead to innovative applications, transforming the landscape of restorative dentistry.

Conclusion

In conclusion, the integration of artificial intelligence into restorative dentistry marks a significant leap forward in enhancing patient care, diagnostic accuracy, and treatment outcomes. As AI technologies continue to advance, dental practitioners must navigate challenges related to data privacy, embrace comprehensive training, and uphold ethical standards. The collaborative synergy between dental professionals and AI experts is crucial for unlocking the full potential of AI in advancing restorative dentistry. Encouraging further research, dialogue, and collaboration will foster a dynamic and innovative future where AI contributes seamlessly to the evolution of dental healthcare.

References

1. Agrawal P, Nikhade P, Nikhade PP. Artificial intelligence in dentistry: past, present, and future. Cureus. 2022 Jul 28;14(7).
2. Ahmed N, Abbasi MS, Zuberi F, Qamar W, Halim MS, Maqsood A, Alam MK. Artificial intelligence techniques: analysis, application, and outcome in dentistry—a systematic review. BioMed research international. 2021 Jun 22;2021.
3. ALBAYRAK B, ÖZDEMİR G, US YÖ, YÜZBAŞIOĞLU E. Artificial intelligence technologies in dentistry. Journal of Experimental and Clinical Medicine. 2021 May 2;38(3s):188-94.
4. Asiri AF, Altuwalah AS. The role of neural artificial intelligence for diagnosis and treatment planning in endodontics: A qualitative review. The Saudi Dental Journal. 2022 May 1;34(4):270-81.
5. Basu B, Gowtham NH, Xiao Y, Kalidindi SR, Leong KW. Biomaterialomics: Data science-driven pathways to develop fourth-generation biomaterials. Acta Biomaterialia. 2022 Apr 15;143:1-25.
6. Benetti AR, Michou S, Larsen L, Peutzfeldt A, Pallesen U, Van Dijken JW. Adhesion and marginal adaptation of a claimed bioactive, restorative material. Biomaterial investigations in dentistry. 2019 Dec 20;6(1):90-8.
7. De Angelis F, Pranno N, Franchina A, Di Carlo S, Brauner E, Ferri A, Pellegrino G, Grecchi E, Goker F, Stefanelli LV. Artificial intelligence: A new diagnostic software in dentistry: A preliminary performance diagnostic study. International Journal of Environmental Research and Public Health. 2022 Feb 2;19(3):1728.
8. Ding H, Wu J, Zhao W, Matinlinna JP, Burrow MF, Tsoi JK. Artificial intelligence in dentistry—A review. Frontiers in Dental Medicine. 2023 Feb 20;4:1085251.

9. El Gezawi M, Wölfle UC, Haridy R, Fliefel R, Kaisarly D. Remineralization, regeneration, and repair of natural tooth structure: influences on the future of restorative dentistry practice. ACS Biomaterials Science & Engineering. 2019 Aug 19;5(10):4899-919.
10. Georgeanu VA, Gingu O, Antoniac IV, Manolea HO. Current options and future perspectives on bone graft and biomaterials substitutes for bone repair, from clinical needs to advanced biomaterials research. Applied Sciences. 2023 Jul 22;13(14):8471.
11. Höland W, Schweiger M, Watzke R, Peschke A, Kappert H. Ceramics as biomaterials for dental restoration. Expert review of medical devices. 2008 Nov 1;5(6):729-45.
12. Huang YK, Hsu LP, Chang YC. Artificial intelligence in clinical dentistry: The potentially negative impacts and future actions. Journal of Dental Sciences. 2022 Oct;17(4):1817.
13. Hussein N. Artificial intelligence in dentistry: current issues and perspectives. Artificial Intelligence and Computational Dynamics for Biomedical Research. 2022 Nov 7;8:229.
14. Joda T, Waltimo T, Probst-Hensch N, Pauli-Magnus C, Zitzmann NU. Health data in dentistry: an attempt to master the digital challenge. Public Health Genomics. 2019 Sep 19;22(1-2):1-7.
15. Kilpatrick, N. M., & Burbridge, L. A. L. (2020). Advanced restorative dentistry. In Oxford University Press. DOI:10.1093/oso/9780198789277.003.0019.
16. Kühnisch J, Meyer O, Hesenius M, Hickel R, Gruhn V. Caries detection on intraoral images using artificial intelligence. Journal of dental research. 2022 Feb;101(2):158-65.
17. McCabe JF, Rusby S. Water absorption, dimensional change and radial pressure in resin matrix dental restorative materials. Biomaterials. 2004 Aug 1;25(18):4001-7.

18. Metsälä E, Henner A, Ekholm M. Quality assurance in digital dental imaging: a systematic review. Acta Odontologica Scandinavica. 2014 Jul 1;72(5):362-71.
19. Moharrami M, Farmer J, Singhal S, Watson E, Glogauer M, Johnson AE, Schwendicke F, Quinonez C. Detecting dental caries on oral photographs using artificial intelligence: A systematic review. Oral Diseases. 2023 Jul 1.
20. Mörch CM, Atsu S, Cai W, Li X, Madathil SA, Liu X, Mai V, Tamimi F, Dilhac MA, Ducret M. Artificial intelligence and ethics in dentistry: a scoping review. Journal of dental research. 2021 Dec;100(13):1452-60.
21. Parhi S, Pal S, Das SK, Ghosh P. Strategies toward development of antimicrobial biomaterials for dental healthcare applications. Biotechnology and Bioengineering. 2021 Dec;118(12):4590-622.
22. Patil S. Artificial Intelligence in the Diagnosis of Oral Diseases. PMC. 2022. Available from: https://www.ncbi.nlm.nih.gov/pmc/articles/PMC9139975/
23. Pethani F, Dunn AG. Natural language processing for clinical notes in dentistry: a systematic review. Journal of Biomedical Informatics. 2023 Jan 7:104282.
24. Ratner BD. Biomaterials science: an interdisciplinary endeavor. InBiomaterials science 1996 Jan 1 (pp. 1-8). Academic Press.
25. Revilla-León M, et al. Artificial intelligence applications in restorative dentistry. PubMed. 2022. Available from: https://pubmed.ncbi.nlm.nih.gov/33840515/
26. Rodrigues JA, Krois J, Schwendicke F. Demystifying artificial intelligence and deep learning in dentistry. Brazilian oral research. 2021 Aug 13;35.
27. Schwendicke FA, Samek W, Krois J. Artificial intelligence in dentistry: chances and challenges. Journal of dental research. 2020 Jul;99(7):769-74.
28. Shan T, Tay FR, Gu L. Application of artificial intelligence in dentistry. Journal of dental research. 2021 Mar;100(3):232-44.

29. Suwardi A, Wang F, Xue K, Han MY, Teo P, Wang P, Wang S, Liu Y, Ye E, Li Z, Loh XJ. Machine learning-driven biomaterials evolution. Advanced Materials. 2022 Jan;34(1):2102703.
30. Tayebi L, Moharamzadeh K, editors. Biomaterials for oral and dental tissue engineering. Woodhead Publishing; 2017 Jul 28.
31. Tuzova L, Tuzoff D, Pulver LE. AI in Dentistry. AI in Clinical Medicine: A Practical Guide for Healthcare Professionals. 2023 May 12:104-16.
32. Urban R, Haluzová S, Strunga M, Surovková J, Lifková M, Tomášik J, Thurzo A. AI-assisted CBCT data management in modern dental practice: benefits, limitations and innovations. Electronics. 2023 Apr 4;12(7):1710.
33. Yamaguchi S, Lee C, Karaer O, Ban S, Mine A, Imazato S. Predicting the debonding of CAD/CAM composite resin crowns with AI. Journal of Dental Research. 2019 Oct;98(11):1234-8.
34. Yilmaz EC. Investigation of two-body wear resistance of composite materials for biomaterial application in oral environment: The influence of antagonist material. Materials Technology. 2020 Feb 23;35(3):159-67.

AI in restorative dentisrty

ORIGINALITY REPORT

8%	7%	4%	0%
SIMILARITY INDEX	INTERNET SOURCES	PUBLICATIONS	STUDENT PAPERS

PRIMARY SOURCES

1	www.mdpi.com<br>Internet Source	1%
2	assets.cureus.com<br>Internet Source	1%
3	www.trendmicro.com<br>Internet Source	1%
4	Talal Bonny, Wafaa Al Nassan, Khaled Obaideen, Maryam Nooman Al Mallahi, Yara Mohammad, Hatem M. El-damanhoury. "Contemporary Role and Applications of Artificial Intelligence in Dentistry", F1000Research, 2023<br>Publication	1%
5	mdpi-res.com<br>Internet Source	1%
6	www.researchgate.net<br>Internet Source	<1%
7	head-face-med.biomedcentral.com<br>Internet Source	<1%

8	byte-project.eu<br>Internet Source	<1%
9	www.irjmets.com<br>Internet Source	<1%
10	epub.ub.uni-greifswald.de<br>Internet Source	<1%
11	journaljpri.com<br>Internet Source	<1%
12	doctorpenguin.com<br>Internet Source	<1%
13	www.jazindia.com<br>Internet Source	<1%
14	eurchembull.com<br>Internet Source	<1%
15	journals.lww.com<br>Internet Source	<1%
16	refubium.fu-berlin.de<br>Internet Source	<1%
17	www.intechopen.com<br>Internet Source	<1%
18	www.jptcp.com<br>Internet Source	<1%
19	Celina Silvia Stafie, Irina-Georgeta Sufaru, Cristina Mihaela Ghiciuc, Ingrid-Ioana Stafie	<1%

et al. "Exploring the Intersection of Artificial Intelligence and Clinical Healthcare: A Multidisciplinary Review", Diagnostics, 2023
Publication

20	Marta Revilla-León, Miguel Gómez-Polo, Shantanu Vyas, Abdul Basir Barmak, Mutlu Özcan, Wael Att, Vinayak R. Krishnamurthy. "Artificial intelligence applications in restorative dentistry: A systematic review", The Journal of Prosthetic Dentistry, 2021<br>Publication	<1 %
21	Tim Eschert, Falk Schwendicke, Joachim Krois, Lauren Bohner, Shankeeth Vinayahalingam, Marcel Hanisch. "A Survey on the Use of Artificial Intelligence by Clinicians in Dentistry and Oral and Maxillofacial Surgery", Medicina, 2022<br>Publication	<1 %

Exclude quotes	On	Exclude matches	Off
Exclude bibliography	On		
